# Phimosis in Children

**DOI:** 10.5402/2012/707329

**Published:** 2012-03-05

**Authors:** Sukhbir Kaur Shahid

**Affiliations:** Consultant Pediatrician and Neonatologist, Shahid Medical Centre, Mumbai-400 077, India

## Abstract

Phimosis is nonretraction of prepuce. It is normally seen in younger children due to adhesions between prepuce and glans penis. It is termed pathologic when nonretractability is associated with local or urinary complaints attributed to the phimotic prepuce. Physicians still have the trouble to distinguish between these two types of phimosis. This ignorance leads to undue parental anxiety and wrong referrals to urologists. Circumcision was the mainstay of treatment for pathologic phimosis. With advent of newer effective and safe medical and conservative surgical techniques, circumcision is gradually getting outmoded. Parents and doctors should a be made aware of the noninvasive options for pathologic phimosis for better outcomes with minimal or no side-effects. Also differentiating features between physiologic and pathologic phimosis should be part of medical curriculum to minimise erroneous referrals for surgery.

## 1. Introduction

“Phimosis” is inability to withdraw the narrowed penile foreskin or prepuce behindthe glans penis [1]. It is a not so uncommon complaint for which a child is brought to office of paediatrician. Parents are often overtly anxious and over-concerned about this nonretractability in their infant or toddler. Most of these cases end up in surgical interventions in form of circumcision. Analyses of medical records carried out in England and Western Australia revealed that medically indicated circumcisions were seven times more than the expected incidence of phimosis in children less than 15 years of age [2, 3]; implying thereby that there is a high rate of unnecessary circumcisions [4]. The operation of circumcision is not devoid of adverse effects and also has a huge economic impact [1, 5–7]. In order to avoid such unindicated expensive operations, it is important to elaborately redefine phimosis and know about newer noninvasive cheaper and safer treatment options.

## 2. Penile Development and Anatomy

Penile formation starts from 7th week of gestation and is complete by 17th week [8]. The integument of the penis in front folds on itself to form the prepuce or foreskin. It covers glans penis and urinary meatus. It serves many functions; the main being protective, immunologic, and erogenous. The inner mucous membrane of this 15 square inches double-layered fold merges with glans [9]. It is bound to under-surface of glans by means of a highly sensitive tissue called frenulum or “little bridle”. Prepuce is richly vascularised and innervated. Fine touch receptors abound on prepuce. Conventional circumcision removes most of these sensitive areas [10]. Unlike the prepuce, glans has only pressure receptors and no fine touch receptors. Glands present on prepuce and glans produce secretions, which aid in lubrication and defence against infection. Lysozyme in these secretions acts against harmful microorganisms. Cathepsin B, chymotrypsin, neutrophil elastase, cytokine, and pheromones such as androsterone are also produced. Langerhans cells are present in prepuce and they seem to provide resistance against HIV infection [9, 11, 12]. At birth and first few years of life, inner part of foreskin is adhered to glans and hence is nonretractile. This separates gradually over time leading to increased retractability.

## 3. Defining Phimosis

Around 96% of males at birth are noticed to have a nonretractile foreskin. This is due to naturally occurring adhesions between prepuce and glans and due to narrow skin of prepuce and “frenulum breve.” This is physiological phimosis. The foreskin gradually becomes retractable over a variable period of time ranging from birth to 18 years of age or more. This is aided by erections and keratinisation of the inner epithelium [13]. Thus preputial retractability improves with increasing age. But 2% of normal males continue to have non-retractability throughout life even though they are otherwise normal [14–18]. In physiologic phimosis, the distal portion of foreskin is healthy and pouts with gentle traction. The narrowed part is proximal to the preputial tip. This differs from pathological phimosis wherein gentle traction leads to formation of a cone-shaped structure with the distal narrow part being white and fibrotic. The meatal opening is also pin-point [4]. It is important to distinguish between these two types of phimosis because their treatment varies widely. Whereas physiological phimosis only needs a conservative approach, surgical management seems justified in pathological phimosis.

But medical doctors are not trained enough to distinguish between these two types of phimosis [3, 19–22]. Their misdiagnosis leads to unnecessary anxiety in parents and over-referrals to urologists for circumcision. Of the cases referred to a urology clinic, it was found that only 8–14.4% of them had “true” phimosis needing surgical intervention [23, 24].

## 4. Etiology of Phimosis

Physiologic phimosis is the rule in newborn males. The prepuce is adhered to glans and this separates over time. Enthusiastic attempts to retract foreskin in physiological phimosiscauses microtears, infection, and bleeding with secondary scarring and true phimosis. Poor hygiene and recurrent balanitis (infection of glans penis), posthitis (inflammation of foreskin), or both could lead to difficulty in retraction of foreskin and consequent true phimosis. Diabetes mellitus predisposes to these infections due to high glucose content of urine, which is conducive for bacterial proliferation [25–27]. Pathologic phimosis may also be due to balanitisxerosisobliterans (BXO), a genital form of lichen sclerosus et atrophicus. This condition affects both men and boys. Its etiology is unknown; infectious, inflammatory, and hormonal causes have been implicated. It may represent a premalignant state [28]. Repeated catherization could also lead to phimosis.

## 5. Clinical Features

The incidence of pathological phimosis is 0.4 per 1000 boys per year or 0.6% of boys are affected by their 15th birthday. This is much lesser than physiological phimosis, which is common in younger children and decreases with age [3]. Physiologic phimosis involves only non-retractability of the foreskin. There may be some ballooning during urination. But pain, dysuria, and local or urinary infections are not seen in these cases. Even if urinary infection is present, it is usually not attributed to the phimosis. On gentle traction, the prepuce puckers and the overlying tissue are pink and healthy. In pathologic phimosis, there is usually pain, skin irritation, local infections, bleeding, dysuria, hematuria, frequent episodes of urinary tract infections, preputial pain, painful erection sand intercourse, and weak urinary stream. Occasionally, enuresis or urinary retention is noticed. The meatal opening is small and the tissue in front of the foreskin is white and fibrotic [29–31]. Phimosis due to BXO is severe with meatal stenosis, glanular lesions, or both [28].

Phimosis in boys and adults can vary in severity. Meuli et al. have graded severity of phimosis into following 4 grades [32], namely, Grade I— fully retractable prepuce with stenotic ring in the shaft, Grade II—partial retractability with partial exposure of the glans, Grade III—partial retractability with exposure of the meatus only, and Grade IV—no retractability. There is another classification of phimosis severity invented by Kikiros et al., which is as follows: Grade 0 is full retractability, Grade 1 is full retraction but tight behind glans, Grade 2 is partial exposure of glans, Grade 3 is partial retraction with meatus just visible, Grade 4 is slight retraction but neither meatus nor glans visible, and Grade 5 is absolutely no retraction [15, 33, 34]. Based on state of the foreskin, phimosis is categorised in order of increasing severity as normal, “cracking,” scarred, and balanitis xerotica obliterans [33, 34].

## 6. Diagnosis

Diagnosis of phimosis is primarily clinical and no laboratory tests or imaging studies are required [35]. These may be required for associated urinary tract infections or skin infections. Treating physician should be able to distinguish developmental non-retractability from pathological phimosis. Grading of severity of phimosis should be done. Determination of etiology of phimosis, if possible, should be tried.

## 7. Management

When a child is brought with history of inability to retract the foreskin, it is important to confirm whether it is physiologic or pathologic. Management depends on age of child, type of nonretraction, severity of phimosis, cause, and associated morbid conditions.

## 8. Reassurance and Vigilance

When it is certain that phimosis in the child is not pathologic, it is vital to reassure the parents on normalcy of the condition in that age group. They should be taught how to keep the foreskin and its undersurface clean and hygienic. Normal washing with lukewarm water and gentle retractions during bathing and urination makes the foreskin retractile over time [36]. Mild soap can be used, but avoid strong soaps as it could lead to chemical irritant dermatitis and further phimosis. Reassurance and reinforcement of proper preputial hygiene may need to be repeated at periodic intervals.

## 9. Topical Steroids

Topical steroids have been tried in cases of phimosis since more than 2 decades. Overall, studies using topical creams for phimosis have yielded dramatic results. Efficacy figures range from 65 to 95% [1]. Mechanism of action of topical steroid therapy in phimosis is not exactly known. It is believed to act via its local anti-inflammatory and immunosuppressive action. Mere moisturising is not the mode of action as prior use of moisturizing agents has failed to yield positive results. Golubovic et al. compared topical steroids with vaseline and found that 19/20 males benefited with steroids, whereas only 4/20 in vaseline group improved [37]. Steroids probably act by stimulating production of lipocortin. This in turn inhibits activity of phospholipase A2 and hence arachidonic acid production is decreased. Steroids also decrease mRNA and hence interleukin-1 formation is diminished. This causes anti-inflammation and immunosuppression [38]. Steroids also cause skin thinning. Dermal synthesis of glycosaminoglycans (especially hyaluronic acid) by fibroblasts is reduced. Epidermal proliferation and stratum corneum thickness is also decreased [39]. Betamethasone 0.05% applied twice a day over a 4-week period has consistently shown good results [13, 34, 37, 40–45]. Success rate was higher in older boys with no infection [40, 43]. Poor compliance was noted to be cause of failures [44]. Studies carried out in younger children have also yielded good results [45]. 0.1% betamethasone cream usage also generated comparable results [46]. Dewan et al. found an efficacy of 65% with 1% hydrocortisone cream [47]. Other steroids tried and found to be effective in phimosis include clobetasol proprionate 0.05%, 0.1% triamcinolone, and mometasone dipropionate [42, 48–53]. The age of the patient, type and severity of phimosis, proper application of the ointment, compliance with treatment, and the necessity of pulling back on the foreskin on a regular basis contribute to either success or failure of the medication [42, 44]. Adverse effects with topical steroids were rare and mild and include preputial pain and hyperemia. No significant side-effects were reported even in young patients [45]. Topical steroids are cheaper than circumcision by 27.4% [7, 54, 55]. They are also less harrowing and devoid of psychological trauma which is commonplace with circumcision [56]. Studies have shown that retractability declines several months after completion of therapy [33, 40]. However, a second course of topical steroids proves useful in such cases. Of concern to parents and providers alike is the degree of risk of systemic absorption of steroid and suppression of hypothalamic-pituitary-adrenal (HPA) axis. But this risk is minor considering the fact that the amount of steroid cream used and surface area of application is small. Besides, steroids are used only for 4–6 weeks. Golubovic et al. found that morning cortisol levels were not significantly elevated in patients who received betamethasone ointment versus controls [37]. Topical steroids could be used as a first-line treatment for pathologic phimosis and is a viable option prior to surgery. However, patients with BXO respond poorly to topical steroids. This may serve as a screening tool in such cases [57].

In order to alleviate the concern over side-effects of topical steroids, nonsteroid anti-inflammatory ointment, diclofenac sodium thrice daily was evaluated and found to be 75% efficacious as compared to petroleum jelly, which was effective in none of the cases used [58]. 0.1% estrogen cream has also been tested and found to be effective in 90% of the cases [59].

If a patient has concomitant balanitis or balanoposthitis, depending on the etiology, he may be treated with topical antibiotics or antifungals [60]. Proper serum glucose control is vital in diabetic patients [61].

## 10. Dilation and Stretching

In this, gentle preputial retractions are carried out by a doctor on an outpatient basis. This nonsurgical adhesiolysis is found to be effective, cheap, and safe treatment for phimosis [23, 62–64]. Eutectic mixture of local anaesthetics (EMLA) could be used prior to attempts at release of the preputial adhesions [65]. He and zhou used a specially designed patented balloon catheter with local anaesthesia in 512 boys and found it to be 100% useful. The technique was simple, safe, cheap, less painful, and less traumatising then the conventional circumcision. It was found to be more beneficial in younger children with no fibrosis or infection [66]. Combination therapy using stretching and topical steroids has also yielded excellent results [67, 68].

## 11. Surgical

These invasive measures are to be reserved for recalcitrant phimosis that fails to respond to medical management.

### 11.1. Conservative Surgical Alternatives

It is a conservative alternative to traditional circumcision which is fraud with many complications, problems, and risk [7, 69–88]. Preputioplasty is the medical term for plastic surgery of the phimotic prepuce. This procedure has faster less painful recovery, less morbidity, less cost, and more preservation of foreskin and its various projectile, erogenous, and sexual physiologic functions [7, 86]. The disadvantage is that phimosis can recur [89]. Dorsal slit with transverse closure is recommended by many doctors due to its simplicity and good results [80]. The lateral procedure described by Lane and South provides cosmesis [85]. Frenulotomy and meatoplasty is also beneficial. Some of the procedures such as Y- and V-plasties (The Ebbehoj procedure) are complex and require skilled hands. Hence they are not favoured much.

### 11.2. Conventional Male Circumcision

In this case, the phimotic foreskin is totally excised. Circumcision is one of the oldest elective operations known in humans. It started as a religious/ritual sacrifice [90]. But gradually it became a routine neonatal procedure in USA and in some countries of Euro pein view of its reported hygiene and cancer-preventing benefits [91]. It cures phimosis and prevents recurrence [92]. It also prevents further episodes of balanoposthitis and lowers incidence of urinary tract infections [26, 93–95]. But it is besot with its own innumerable short, and long-term problems. Pain, difficult recovery, bleeding, infection, psychological trauma, and high cost are seen with circumcision [96, 97]. The literature is full of reports of morbidity and even deaths with circumcision. Besides, circumcision could lead to keloid formation. Possibility of decline in sexual pleasure for both circumcised males as well as their female partners due to loss of erogenous tissue has been reported [96, 98–105]. With advent of newer plastic surgical procedures for phimosis, this traditional surgery is gradually getting outdated. Circumcision is to be avoided in children with genital anomalies where the foreskin may be needed for later corrective surgery for the anomaly.

## 12. Other Experimental Options

Prolonged antibiotic therapy, intralesional steroid injection, carbon dioxide laser therapy, and radial preputioplasty alone or with intralesional injection of steroid have all been described as therapies for phimosis, but there are no proper randomised controlled trials of their efficacy and long-term outcomes.

## 13. Summary

Phimosis needs to be differentiated from non-retractile prepuce, which is the rule in young children. Doctors should be taught on distinguishing these two types of phimosis in order to avoid parental anxiety and needless referrals to urologists for circumcisions. Newer nonsurgical modalities such as topical steroids and adhesiolysis are effective, safe, and cheap for phimosis in children. Parents should be made aware of these measures to treat phimosis. If surgery is indeed needed, conservative plastic surgical techniques should be performed rather than the traditional circumcision. This would help the patients, their family, and the healthcare as well as the society at large.
